# Exploring survivor perceptions of pre-eclampsia and eclampsia in Nigeria through the health belief model

**DOI:** 10.1186/s12884-019-2582-2

**Published:** 2019-11-21

**Authors:** Pooja Sripad, Karen Kirk, Gloria Adoyi, Amy Dempsey, Salisu Ishaku, Charlotte E. Warren

**Affiliations:** 1 Population Council, 4301 Connecticut Avenue NW Suite 280, Washington, DC 20008 USA; 2 Population Council, One Dag Hammarskjöld Plaza, 3rd Floor, New York, NY 10017 USA; 3 Population Council, No. 16 Mafemi Crescent, Utako District, Abuja, Nigeria; 4 Julius Center for Health Science and Primary Care, University Medical Center, Utrecht University, Utrecht, Netherlands

**Keywords:** Maternal health, Community awareness, Pre-eclampsia, Care-seeking behavior, Health belief model

## Abstract

**Background:**

In Nigeria, hypertensive disorders have become the leading cause of facility-based maternal mortality. Many factors influence pregnant women’s health-seeking behaviors and perceptions around the importance of antenatal care. This qualitative study describes the care-seeking pathways of Nigerian women who suffer from pre-eclampsia and eclampsia. It identifies the influences – barriers and enablers – that affect their decision making, and proposes solutions articulated by women themselves to overcome the obstacles they face. Informing this study is the health belief model, a cognitive value-expectancy theory that provides a framework for exploring perceptions and understanding women’s narratives around pre-eclampsia and eclampsia-related care seeking.

**Methods:**

This study adopted a qualitative design that enables fully capturing the narratives of women who experienced pre-eclampsia and eclampsia during their pregnancy. In-depth interviews were conducted with 42 women aged 17–48 years over five months in 2015 from Bauchi, Cross River, Ebonyi, Katsina, Kogi, Ondo and Sokoto states to ensure representation from each geo-political zone in Nigeria. These qualitative data were analyzed through coding and memo-writing, using NVivo 11 software.

**Results:**

We found that many of the beliefs, attitudes, knowledge and behaviors of women are consistent across the country, with some variation between the north and south. In Nigeria, women’s perceived susceptibility and threat of health complications during pregnancy and childbirth, including pre-eclampsia and eclampsia, influence care-seeking behaviors. Moderating influences include acquisition of knowledge of causes and signs of pre-eclampsia, the quality of patient-provider antenatal care interactions, and supportive discussions and care seeking-enabling decisions with families and communities. These cues to action mitigate perceived mobility, financial, mistrust, and contextual barriers to seeking timely care and promote the benefits of maternal and newborn survival and greater confidence in and access to the health system.

**Conclusions:**

The health belief model reveals intersectional effects of childbearing norms, socio-cultural beliefs and trust in the health system and elucidates opportunities to intervene and improve access to quality and respectful care throughout a woman’s pregnancy and childbirth. Across Nigerian settings, it is critical to enhance context-adapted community awareness programs and interventions to promote birth preparedness and social support.

## Background

Despite progress made in maternal health and shifting mortality patterns, evidence shows growing disparity around access to quality maternal care. It is essential to place perspectives, wants, and needs of individuals who seek and provide maternal health services at the center of shaping strategies to promote access to live-saving care [1].

*“The true engine of change in maternal health…will be the determination of people at the front-lines of health systems – patients, providers, and managers – to find or take the power to transform their own lived reality. Our job in global health is first to listen to them, and then to co-create the conditions at every level of the system that can make that locally drive transformation possible.”* [1]

In a world where 830 women die every day from pregnancy- or childbirth-related causes, persisting causes of death among women of reproductive age are hemorrhage (18%), unsafe abortion (18%), hypertensive disorders (12%), sepsis (9%), and other maternal disorders (22%). Ninety-nine percent of these deaths occur in low- and middle-income countries and are more common among poor, rural women than urban women [2, 3]. Preventing these deaths requires deeper investigation into the contributory causes of maternal mortality, the quality of care women receive, and their experiences during labor and delivery at health facilities.

In Nigeria, hemorrhage and pre-eclampsia/eclampsia make up over 50% of the country’s maternal mortality. With strides in preventing hemorrhage-related deaths, hypertensive disorders have become the country’s leading cause of maternal mortality, accounting for 29% of these deaths in tertiary hospitals [4]. A study conducted in Ogun State, Southwestern Nigeria found that location, time, obstetric condition, and socio-cultural characteristics influence health-seeking behaviors among pregnant women [5]. This study also showed that while some women preferred accessing health services at facilities, most preferred traditional doctors, healers, religious care, and traditional birth attendants during pregnancy and delivery. A lack of financial resources further delays seeking care, as women depend on their husbands to provide money for health services. Family members play a significant role in women’s ability to access health care during pregnancy [6]. Since families consider pregnancy to be a natural part of life, they underestimate the importance of antenatal care (ANC) and the severity of danger signs and symptoms of pregnancy-related complications. Despite their social proximity to a care seeking woman, limited qualitative research exists on the influence of family members –spouses, parents, guardians and others – and community members on maternal care-seeking decisions in Nigeria.

### Health belief model

The health belief model is a cognitive value-expectancy theory that provides a framework for understanding women’s perceptions of pre-eclampsia-related care seeking. In linking socio-demographics to perceptions and cues to action, the health belief model asserts that beliefs and attitudes predict health-seeking behaviors. The health belief model enables us to understand perceived susceptibility and perceived severity or threat of a specific health problem as well as the perceived benefits of and perceived barriers to its recommended solution [7]. It also considers ‘cues to action’ and the notion of self-efficacy in decision-making around a specific behavior. In many cultures, Nigeria included, pregnancy is a shared experience by a woman and her family. Studies that apply the health belief model to understanding women’s actions during pregnancy explore healthy eating and being physically active as behaviors of focus and suggest that women’s knowledge of what to do during pregnancy influences their perception of severity/threats of health risks [8, 9]. In the scenario of maternal health, pregnant women are more likely to engage in a specified behavior when perceived benefits override barriers. To our knowledge, the health belief model has not been used to look at pre-eclampsia and eclampsia nor to understand socio-cultural influences in Nigeria.

This study describes the care-seeking pathways of Nigerian women who suffer from pre-eclampsia and eclampsia. It identifies the influences – barriers and enablers – that affect their decision making, and proposes solutions articulated by women to overcome the obstacles they face.

## Methods

This study adopted a qualitative design that fully captures the narratives of women who experienced pre-eclampsia and eclampsia during their pregnancy. Data were collected over a five-month period from April to August 2015 in seven states across Nigeria: Bauchi, Cross River, Ebonyi, Katsina, Kogi, Ondo and Sokoto. Selected states include representation from each geo-political zone, covering the cultural diversity, varied socioeconomic development and differential access to health care services.

Forty-four individual in-depth interviews (IDIs) were conducted with women who experienced pre-eclampsia, purposively selected and recruited in the community through health facility referrals by community health extension workers. Participants were identified as survivors of pre-eclampsia by health care providers who managed – provided some level of care and referral – during women’s labor and deliveries. While survivors likely experienced danger signs, they all received care either during ANC, childbirth, and/or in the early postpartum period. Eligibility criteria was not restricted to women who delivered in facilities nor differentiated on progression to eclampsia prior to care-seeking. The data collection team used a contact tracing approach and engaged local guides to recruit women and interview them in the community. Data collectors experienced in qualitative methods and reproductive health were trained on study topics, interview guides, and research ethics. Data collectors had no prior established relationship with study participants. Instruments were pre-tested during the training. Women were asked open-ended questions related to their pregnancy, delivery, and postnatal experiences using a structured guide (Additional file 1) that included probes on quality of ANC, knowledge of pre-eclampsia and eclampsia, and factors that affect care seeking at individual, household, community and health systems levels. Socio-demographic information was collected to contextualize our findings. After obtaining written informed consent from participants, data collectors conducted interviews in local languages including Hausa (Bauchi, Katsina, Kogi, and Sokoto), Yoruba (Ondo), Igbo (Ebonyi), and Ibibio (Cross River). Care was taken to interview women in private settings. Two people attended each interview; one conducted the interview and the other took field notes.

Interviews were audio-recorded, transcribed verbatim and translated into English. With a grounded theory orientation, after an initial reading of the transcripts, a code structure was inductively developed, discussed, and applied to the data using the NVivo 11 qualitative software by two researchers. Memos written while coding the data allowed researchers to describe similarities and differences in women’s perspectives between states, by age of marriage and parity, as well as emergently relevant characteristics. Through a deliberative process, researchers further grouped codes into themes and analyzed the local findings in dialogue with the health belief model.

Ethical approval for this study was granted by the Population Council’s institutional review board (Protocol #693), the National Health Research Ethics Committee of Nigeria, and research ethics committees from each study state.

## Results

The 42 women interviewed were aged 17–48 years (median age: 30 years), married on average around the age of 21 years (range 13–37 years), and had between 0 and 8 children (Table 1). Women who married under 18 years of age (or ‘early’) lived in the northern states (Bauchi, Katsina and Sokoto) where the median age at marriage among our sample was 17 years (range: 13–25 years). Of the 27 women who provided information on their level of education, 7 did not attend any school, 3 completed primary, 8 completed secondary, and 9 completed a higher degree (associates, bachelors, masters). The majority (39/42) of respondents attended ANC for at least one of their pregnancies. Those who did not attend ANC were teenage (aged 17–19 years) or older (36 years) mothers. The 32 respondents whose narratives approximated timing of first ANC visit during their recent pregnancy sought services at various stages (*n* = 12, *n* = 15, *n* = 5 in the first, second and third trimesters, respectively). Women who only received ANC in their third trimester were young (4/5 were 17–20 years). Out of the 42 women who experienced pre-eclampsia, 18 developed eclampsia and 8 experienced perinatal death. Out of the 18 women who developed eclampsia - often reporting loss of consciousness as proxy for a seizure, 8 recalled it occurring at home, 2 mentioned during transit or in a village setting, 4 described it taking place at a hospital, and 6 did not specify.

**Table 1:** Characteristics of Women Who Experienced PE/E

	Northern States	Southern States	Total
Age at interview			
< 17	5	0	5
18–29	11	2	13
30–48	6	16	22
Missing	1	1	2
Age at marriage			
< 17	10	1	11
18–29	9	10	19
30–37	1	5	6
Missing	3	3	6
Education			
None	7	0	7
Primary	3	0	3
Secondary	4	4	8
Higher Degree	2	7	9
Missing	7	8	15
Number of Children			
0	4	1	5
1–2	10	8	18
3–4	5	6	11
5+	3	2	5
Missing	1	2	3
Age of youngest child			
< 1 year	9	5	14
1–5 years	4	3	7
5+ years	0	4	4
Missing	10	7	17
Birth Outcome			
Alive	17^^^	14	31
Perinatal Death	4^^^	4	8
Missing	4	1	5

^^^Two cases of twin births where one baby survived, and one died

**Fig. 1:**
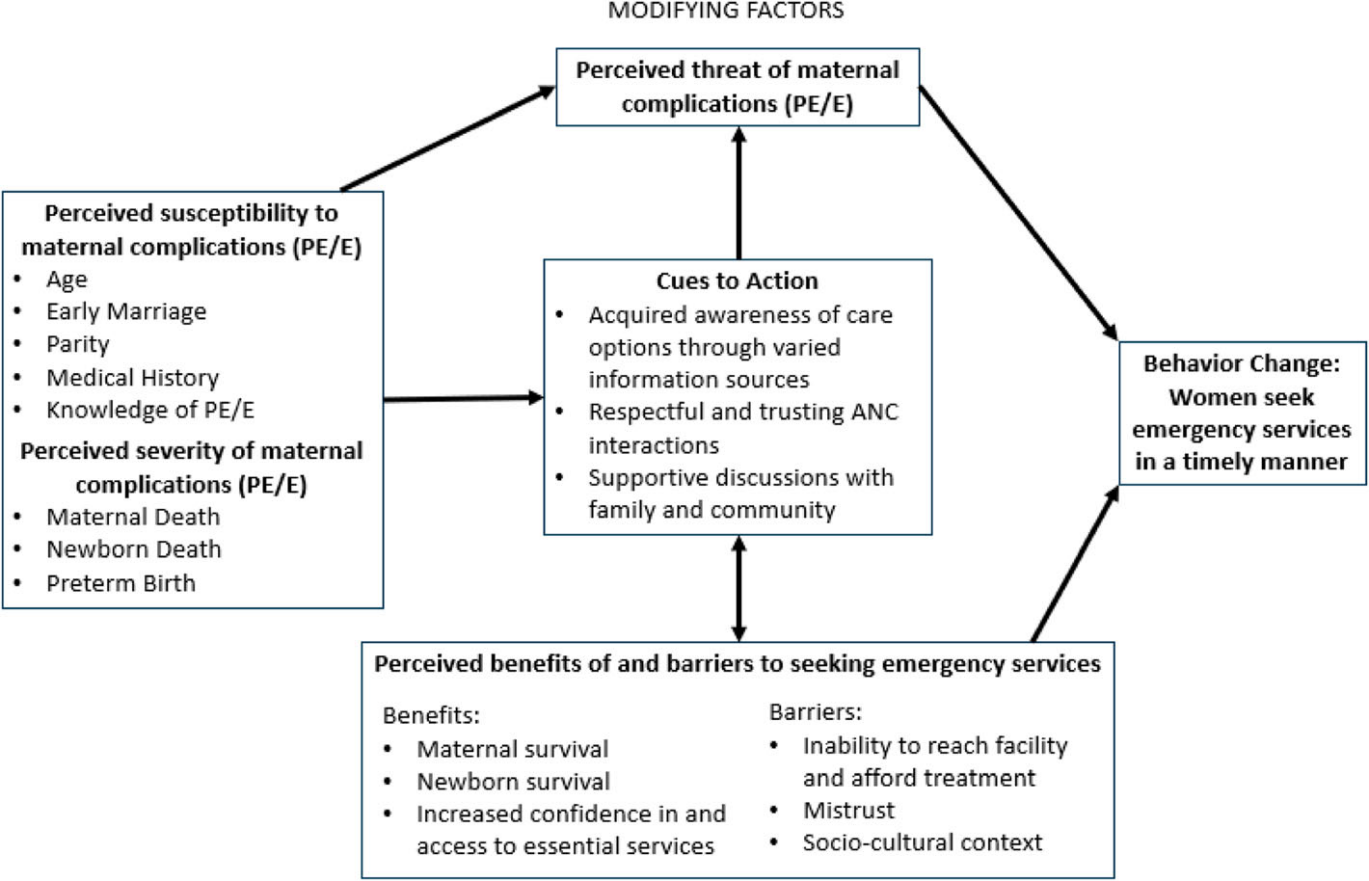
Nigerian women’s perspectives on care-seeking for pre-eclampsia and eclampsia applying a modified health belief model.*Adapted from (Champion & Skinner, 2008)

We found that many beliefs, attitudes, knowledge and behaviors of women are relatively consistent across the country, with some variation among those in the north who married and became pregnant early. Nigerian women’s perceptions of the susceptibility and threat of health complications during pregnancy and childbirth, including pre-eclampsia and eclampsia, were moderated by a number of factors including individual characteristics, medical histories and knowledge of causes, cues to action (awareness to seek care, trust in the health system, and family and community support), as well as perceived barriers (mistrust of health systems, socio-cultural barriers) and benefits (maternal/newborn survival) to seeking timely care influence care-seeking behaviors (Fig. 1).

### Perceived susceptibility, severity and threat

Women’s perceived susceptibility of maternal health complications (e.g. pre-eclampsia/eclampsia) reflects their sense of risk – namely, their internal calculation of how likely they are to suffer complications. Perceived severity of these complications includes maternal and newborn death, as well as preterm birth; the combined notions of susceptibility and severity capture women’s perceived threat of pre-eclampsia and eclampsia and its consequences. Though women’s narratives suggest perceived susceptibility are often influenced by age, early marriage and/or parity, knowledge, and personal or family medical histories, a prevailing lack of comprehensive knowledge on pre-eclampsia and eclampsia (including danger signs and symptoms, causes, management, counseling, health risks during and after pregnancy) emerged across all states.

#### Individual characteristics, histories and knowledge of causes and implications

Variable knowledge of the causes of hypertension in pregnancy persist. Most women describe pre-eclampsia and eclampsia as brought on suddenly by stress (stress caused by household problems, normal activity, or pregnancy) or by ‘*thinking too much’*; however, quite a few women report not knowing about these complications and related implications for maternal and newborn health outcomes.

*“The first time I registered for antenatal I was told my BP [blood pressure] was too high so I never thought it was a problem, I thought it was a normal stress or maybe it was due to stress during pregnancy…nobody told me that it was a problem.”* (Ondo, 2 children)

Early marriage, common in the north (e.g. Katsina), appears associated with less comprehensive knowledge of causes and implications of pre-eclampsia and eclampsia – potentially tied to women’s limited educational status. In contrast, in the south (e.g. Cross River), women describe the notion of spiritual interference and witchcraft as a cause of pre-eclampsia and eclampsia.

*“When I first took - in [conceived] this time around, there was no problem just like in the case of other normal pregnancies. I thought it will equally go well, but to my surprise, things started changing as the pregnancy got older. I started with fever, later headache and dizziness. I did not go to hospital or ANC yet*.” (Katsina, 5 children)

*“Sometimes they will tell you it’s spiritual. They will tell you, a pregnant woman is supposed to be like this, be like that. Seek spiritual attention from traditional whatever...”* (Cross River, 4 children).

In all states, one’s own medical and family history as well as exposure to ANC messaging significantly modified perceptions of susceptibility and threat of incurring pre-eclampsia and eclampsia as a complication and risk of maternal death. Women who previously experienced – or had a family member who experienced – pre-eclampsia and eclampsia during childbirth were primed in perceiving the severity of complications that manifest as a real threat compared to first time pregnant women.

*“My mom died of high blood related illness, […] I remember when I was a kid, my mother during her sixth pregnancy was operated upon, and she lost the baby and was unconscious for some time and after that she couldn’t take in [conceive] again. I don’t know whether it was [high BP] or pre-eclampsia. Eventually she died of hypertension. My immediate younger sister had the same problem before she took in [conceived] and during pregnancy though she delivered safely through cesarean section.”* (Cross River, 2 children)

As the 40-year old woman with two children recounts, her perceived susceptibility to pre-eclampsia and the severity of negative consequences drew primarily from her family medical history. This woman went on to attend ANC and access emergency obstetric care where she delivered by cesarean section. She also reflected on her own personal history of hypertension in pregnancy.

*“In the third pregnancy, the same thing occurred. My BP went up again in the first trimester and I went to the hospital early and was placed on drugs, nifedipine and aldomet and it went down a bit, but later went up and I lost the pregnancy. The experience of losing the pregnancy even triggered it the more…the fourth time I registered early because of the high BP problem.”* (Cross River, 2 children)

Most women who attended ANC for one child continued to do so for subsequent pregnancies and had greater knowledge of pre-eclampsia and eclampsia, suggesting that ANC plays a critical role in birth preparedness, threat comprehension, and care seeking for obstetric emergencies.

### Cues to action

Cues to action on the perceived threat are broadly situated in enabling events or discussions that influence care seeking for pregnancy complications, including pre-eclampsia and eclampsia.

#### Acquired awareness of care options

Information sources for women on maternal complications and comprehension of danger signs and symptoms of pre-eclampsia and eclampsia – including the engagement of PHC providers, such as community health extension workers that helped with participant recruitment and provided ANC – emerge as cues to action. Comparing narratives from the south and north, we found that irrespective of education status, age of marriage, parity, and ANC attendance, women maintained similarly limited awareness of the signs and symptoms of pre-eclampsia and eclampsia, a perceived barrier to care seeking. This widespread lack of awareness points to concerns around quality and content of ANC messaging on clinical manifestations of maternal complications and overall birth preparedness. Despite generally knowing that high blood pressure is a potential problem during pregnancy, few women fully understood the significance of their own hypertension and the subsequent risk to their labor and delivery. Similarly, they did not comprehend the possible severity (i.e. maternal/newborn death). The following examples show how awareness of pre-eclampsia and eclampsia affects the quality of ANC interaction and learning; the first woman describes her inability to ask the right questions to health workers during ANC and the second describes the need for awareness-building, through media, community-focused intervention, and PHC provider-provided ANC as a cue to action.

*“I did not attempt asking it [effect of high blood pressure] because I didn’t know things of this nature [implications of BP] can come up.”* (Kogi, 4 children)

*“Awareness is very important; if awareness is created through the media, and when we are talking about pre-eclampsia/eclampsia, it should be in simple language that the common person understands* […] *When they go for ANC, women should ask the care givers of drug to take to prevent high BP especially those that have a history of high BP in the family… If pregnant women are enlightened that when they go for ANC, they should ask the nurses, ‘since my mother had these issues, is there anything you can give me so that I will not develop the same problem?’”* (Cross River, 2 children)

Mixed quality of care at ANC in each state affects the extent to which women with pre-eclampsia/ eclampsia know where and when to seek emergency obstetric services. Though most women said that their blood pressure was taken, or urinalysis conducted, providers rarely explained results and implications. Often women describe having been prescribed blood pressure or other routine medications (e.g. antihypertensive, paracetamol, malaria) without adequate explanation. Consequently, not all women attributed their pregnancy risk to high blood pressure. No participants describe having abnormal urine – urinalysis results in relation to pre-eclampsia/eclampsia was never followed up during provider-patient discussions.

#### Respectful and trusting ANC interactions

Women’s descriptions of their ANC and delivery experiences related to the technical quality of health services and respectful and trusting maternity care interactions, a cue to care-seeking action. Some women spoke about available (free) essential drugs for high blood pressure and supplies for safe delivery in government facilities, precursors to quality of care and trust. However, as alluded to in the prior sub-section, when women did not receive adequate explanation about their condition or about any prescribed medications including potential side effects, their trust waivers, and cues to action (management of condition and care-seeking in the future) are jeopardized.

*“In the hospital, they advise and prescribe drugs for patients. But some patients tend to stop taking the drugs because of some side effects experienced… some don't buy the drugs. Usually, hypertensive drugs cause different things, if you don't tell what the drug reactions are, they wouldn't know. So, some decide to stop the drugs without coming back to hospital to explain what they feel when the drugs are swallowed.”* (Sokoto, no child survived)

Respectful maternity care affects care seeking for ANC and delivery as women fairly consistently describe positive experiences influencing their behavior.

*“Then, the nurse that took my labor I would say she was an angel, God sent. She was just too nice, and she delivered my baby safely. I went home and I never thought it was a problem*.” (Ondo, 2 children, 41 years)

*“They attend to me, my first day, they attended to me very well. The second attempt they [also] attended to me very well. I went there, and my BP was high. I needed and asked where I can lay my head and gather myself. They later attended to me.”* (Ebonyi, 7 children)

Women’s narratives suggest that their trust in the health system’s ability to manage maternal complications shifts over time and is determined by internalizing one’s own experience during ANC, delivery and postnatal care as well by learning from others’ experiences. Socio-cognitive processing of a woman’s positive interaction with health providers and systems over the course of her pregnancy, including, receiving appropriate, timely, and respectful, non-judgmental services enhances trust and motivates care seeking. When women have or hear about negative experiences at health facilities, their trust in the system breaks down and delays their decision to seek emergency obstetric care. The following example describes a scenario in which a woman’s perception rooted in community norms and perceptions shifted with her own care experience.

*“Since I have been coming [to ANC], this caring helped save the baby and prevent me from this suffering. We were thinking everything is too costly and the staff might abuse us, but this is not what I found here.”* (Sokoto, 1 child)

This woman’s shift toward respectful and trusting patient-provider relationships suggests that despite her and her husbands’ perceived barriers to care – unfriendly providers and high cost – positive and caring interactions play a role in changing attitudes.

#### Supportive discussions with family and community

Engaging in discussions with family and communities emerged as a critical cue to action as these groups often provide instrumental support in women’s ANC and delivery care seeking. Most women describe interactive discussion-based decisions with their spouse during pregnancy (before childbirth), suggesting husbands have significant influence on whether a woman gets timely care. Mothers-in-law and others in the household were also influential – in the cases of delivery, most women delayed care seeking to till they passed out and a family member (spouse, uncle, mother, father, or sibling) brought them to the facility for care. Some women spoke of health providers and community members as influential, but less so than immediate family. Two women in northern states Bauchi and Katsina decided on their own to seek care.

*“My husband used to tell me to relax and that with time it would go. My mother-in law sometimes encouraged me and sometimes she would talk as if I’m trying to be lazy but good friends would encourage me to always visit my doctors for check-up and other treatment.”* (Ebonyi, 1 child, 31 years)

*“They prescribed some drugs; we bought them even though they were bit expensive. Some my husband bought outside the hospital.”* (Sokoto, no children/currently pregnant)

Some believed that communities and churches ought not to bear influence since maternal complications relate to the private sphere of family decision-making, while others felt they should play a role in motivating timely care seeking and promoting ANC in addition to offering healing prayers.

### Perceived barriers and benefits to seeking timely care

Perceived barriers to seeking timely care include an inability to reach facilities and afford treatment, mistrust of the health system, and socio-cultural context that affect a women’s decision-making to seek emergency obstetric care, considering the perceived threat of complications.

#### Inability to reach a facility and afford treatment

Though not unique to these women or this context, inadequate transportation options and significant costs emerged as significant transportation and financial barriers. In contrast to some women who described living near facilities, many lamented that long distances, challenging terrain, and inability to access vehicles prevent women from seeking services.

*“The distance from the house to where the hospital is, is far, then I was ready to go to that hospital because of the distance, I was not able to make it, I delivered in the house.”* (Kogi, 4 children)

*“[…] later the severe headache I was having surfaced again. I was unconscious, and my husband was out of town, there was no vehicle to convey me to the hospital. I was lucky to be conveyed by a government driver who was coming back from the farm.”* (Bauchi, 8 children)

Financial barriers affect access to transportation and affordability of health services or medicines. Some women in Bauchi, Sokoto, Kogi, Katsina, and Ebonyi experienced financial difficulty in securing transportation to the health facility while others felt that the prescribed drugs or necessary services were expensive. Women in Cross River did not experience the same financial burden for health services, noting that the drugs were affordable, and treatment was provided free of cost.

*“One day when the pregnancy was about 9 months, fever gripped me with severe headache, my body started shaking, I did not go out, I send for my husband, he came and said he will look for money to take me to the hospital, after some time, back pains started and I don’t really know what was happened again.”* (Katsina, 5 children)

*“We have no vehicles around and no money to travel to the Hospital. My husband suggested I went on my foot to the maternity near our village, I did*.” (Bauchi, 4 children)

#### Mistrust

Mistrust of the health system is often rooted in disrespect and abuse during childbirth either experienced or heard about in a community. Harsh or dismissive attitudes of providers toward women (self or others) in the maternity ward, coupled with negative childbirth outcomes – as often occurs with women who experience pre-eclampsia and eclampsia – may perpetuate mistrust of providers and the health system more broadly.

*“80% of nurses are hostile. They talk anyhow, they don't count you as somebody. During my delivery, I went through hell...”* (Ondo, 2 children)

*“The doctor or the nurse would be quarrelling that they don’t want to see her [laboring woman]. […] The doctor […] said that the woman is not yet ready to deliver, that she should go and walk around… the woman said that this baby is coming down and they force the woman to go out of the labor ward to go and climb steps. In the process of climbing that step, the baby fell out and broke his head […] before the woman could know, the baby is already dead. […] the mother was crying the other nurse came and tell her to pay them their bill. Bill for what? For the dead child?”* (Ebonyi, 2 children)

#### Socio-cultural context

While spirituality and tradition affect women’s lives profoundly, they rarely factored into our sample’s actual care-seeking decisions. Women often (particularly in the south) describe the importance of offering prayers (by a third party) to promote well-being of a woman experiencing pre-eclampsia.

*“…despite that God is helping us we depend on that doctor…”* (Ebonyi, 2 children)

One woman described going to a traditional birth attendant and few mentioned using traditionalist/herbalists, but the majority did not use or even mention them.

*“I never attended antenatal care throughout my pregnancies including this recent one. In this current pregnancy, I was well from the beginning until when it was six months when I began to have severe headache few days later, I noticed that my legs had swollen up. They gave me some herbs, the headache goes and comes back, it did not stop completely*.” (Sokoto, 3 children)

*“The church believes everything is a spiritual attack and as a result, they keep you there until you pass out.” (*Cross River, 2 children)

As the above quotes suggest, confusion around spiritual and biomedical causes of complications alongside norms of using traditional remedies may delay necessary care seeking of women experiencing danger signs and symptoms. These complex socio-cultural dynamics manifest through interactions of church and community with families and spouses as primary decision-makers.

### Perceived benefits influence care-seeking behaviors

The perceived benefit of a positive childbirth outcome (maternal and newborn survival) reflects the end goal for survivors of preeclampsia and eclampsia and requires balancing barriers with motivating cues to action, that enable women’s care seeking. Our sample of women who experienced pre-eclampsia consistently describe the outcome benefits of accessing skilled care, though some articulate the merits to continuity of care as a part of developing trusted relationships that would enable better recognition and management of complications.

*“My mind was telling me that if I went to that hospital [where] I delivered before, I won’t have had such problem […] I’m advising other women […] please register in a hospital early as they notice they are pregnant and […] stick to that particular place so that the doctors who started the antenatal would know the situation of their delivery and if there is a problem. They should tackle it at early stage […] Both mother and child would come out alive and healthy.”* (Ebonyi, 2 children)

Survivors also describe candidly an elevated confidence in the health system’s ability to care for them in an emergency as perceived benefit of care seeking – particularly as women receive ANC earlier and are empowered by programming that increases their knowledge of danger signs and symptoms of pre-eclampsia and eclampsia. Survivors also suggest a forward-looking community benefit of increased access to essential emergency services, if community-level education and mobilization.

*“At the community level, I think we need to do more awareness programs for patients so that if they have hypertension, they don’t play with it. It’s not only eclampsia that it leads to.*” (Kogi, 4 children)

## Discussion

Dialoguing Nigerian women’s narratives through a modified health belief model reveals how perceived susceptibility to pregnancy complications relates to perceptions of severity or threat of pre-eclampsia and eclampsia and subsequent care-seeking behavior. Our data also show how this pathway is influenced by a range of modifying factors, including cues to action, and perceived barriers to and benefits of seeking emergency care. Findings indicate that enhancing women’s and community knowledge of pre-eclampsia and eclampsia, danger signs in pregnancy, including supporting social responsiveness to symptoms and enabling access to care, advocating for early-stage interaction with health providers – particularly among younger primigravida women, and strengthening quality ANC interactions between women and providers may lead to positive care-seeking behavior and improved maternal and newborn health outcomes in Nigeria. These intervening points may increase trust and confidence in health systems. Relatively consistent findings across the north and south further suggest that these propositions can be leveraged as a guide and customized to specific gaps in a programming context.

Beyond the minor differences in perceived susceptibility and threat across respondents’ age, other factors including age at marriage, parity, family medical history and knowledge levels (ANC and community-derived) strongly affected women’s perception of pre-eclampsia and eclampsia. By linking prior pregnancies and others’ experiences of pregnancy in which high blood pressure was a concern, respondents illustrate the social learning pathway that occurs around risk, consequences, and care options for pre-eclampsia and eclampsia. The low to moderate quality of ANC (insufficient explanation of clinical implications of blood pressure tests and urinalysis) and birth preparedness (what to do/where to go when danger signs experienced) described by almost all the women regardless of parity, age, or geographic region, triangulate this awareness gap around pre-eclampsia and eclampsia. Spirituality plays a larger role in the South, early marriage confounds associations in the North, and women in Cross River state (South) face fewer financial and transport barriers. (Mis)trust in health systems – including positive and negative interpersonal relationships with providers during ANC and childbirth that may extend into the postpartum period – and social support moderate women’s experiences by affecting whether and how women weigh and act upon barriers and benefits to seeking emergency care.

### Gap in awareness

Knowledge of causes and consequences of pre-eclampsia and eclampsia, in addition to awareness about when and where to seek care, affect a woman’s perception of threat of developing pre-eclampsia as seen in our study and elsewhere. A study conducted in Sokoto, Nigeria interviewed ‘relations’ of women with eclampsia and found that only 7 % of 159 interviewees correctly associated eclampsia with high blood pressure. Instead, they cited ‘evil spirits’, pregnancy, and God as causes and tended to seek traditional rather than biomedical treatment [10]. Another community-based survey collating binary responses from 200 adult women in Tanzania demonstrated the sample’s significant lack of awareness (50% or less across all knowledge items); only 13% knew epigastric pain to be a symptom of pre-eclampsia and half believed evil spirits and exposure to fire are contributory causes of pre-eclampsia [11]. A systematic review confirms that traditions, cultural beliefs, and social norms are common factors that affect perceptions of antenatal and obstetric care in sub-Saharan Africa [12]. It is critical to address this awareness gap and focus on how women are getting their maternal health information. Improving community awareness and understanding of pregnancy risks will allow them to perceive threat of pre-eclampsia and eclampsia and promote timely and appropriate care seeking.

### Inequitable financial burden

Frequently, financial barriers affect access to transportation, availability of drugs, and affordability of services. Many women did describe costs to affect their ability to access care. Respondents from Cross River consistently received free services or paid nominal amounts for their care, possibly related to the public-private partnership, Saving Mothers Giving Life, that works to strengthen the health system and improve maternal and newborn health outcomes [13]. Though Nigeria has made efforts to set up a National Health Insurance Scheme, there has been inconsistent success in reducing out-of-pocket expenditure.

### Trust and experience

The modifying influence of trust and positive experience in contrast to mistrust and negative experience described by Nigerian women is critical to improving the continuum of care received throughout pregnancy (ANC and labor and delivery), particularly in the timely management of complications like pre-eclampsia and eclampsia. The way in which trust and mistrust in the health system motivates and reinforces care-seeking are growing areas of investigative interest in sub-Saharan Africa and in maternity care specifically [14, 15]. Globally, the recognition that trust or confidence in a health system is part of perceived health care quality, further emphasizes the need to understand the mechanisms and measurement of these concepts in practice [16]. The experience of hostile and dismissive attitudes shown by health providers in our study echoes forms of disrespect and abuse described elsewhere in Nigeria [17]. While quality of ANC in Nigeria remains limited and non-comprehensive [18], more research is needed around how trust (and mistrust) interact with experience and outcomes of antenatal, delivery, and postnatal care in Nigeria.

### Limitations

The analysis only considered the perceptions of women who experienced pre-eclampsia. The study team was therefore unable to draw comparisons between those who did and did not experience pre-eclampsia regarding their perceived susceptibility, perceived threat, cues to action, perceived benefits and timely care seeking. However, as maternal recall among women who suffer complications may be similar or higher compared to a general population, the findings maintain credibility. Another limitation was insufficient probing into select themes of spirituality and prayer and disrespect and abuse. Articulation of nuanced mechanisms through which these concepts affected care seeking would have allowed for discerning religious reasons or specific traditional practices leading to delays in care-seeking decisions and behavior among women who experienced pre-eclampsia/eclampsia (and/or their families). All the narratives could have probed further into the context, type, frequency, and determinant of the disrespectful, abusive, or respectful experiences during ANC and labor and delivery.

The health belief model helps explicate what factors motivate individuals to engage in a specific action, it limits commentary to that one action, as opposed to distinguishing future intentions and behaviors. Influence of social norms and the gap between intent and actual behavior change cannot be fully captured in health belief model but arise as an issue in Nigeria. Future research should consider the Theory of Normative Social Behavior [19] or similar models that consider the influence of descriptive and injunctive norms and interpersonal communication on individual’s health-seeking behavior.

### Implications

Despite these limitations, study findings reinforce a need to address educational deficiencies, health care attitudes, and access to resources at the community level to effectively harness cues to action and mitigate the barriers faced by women to seek care. Connecting women with timely care in this context requires:

- Increasing awareness of hypertension in pregnancy through community-based health promotional activities that leverage locally relevant and trusted forums;
- Improving quality of ANC at primary health care level, closer to where women live; and
- Strengthening primary health care givers’ ability to manage hypertension in pregnancy and make timely referrals to hospitals.

Community-focused interventions that emphasize birth preparedness for all women during their pregnancy and adopt social behavior change approaches render cues to action (in case of a complication) more interpretable [20]. In Nigeria, the role of communities and social leaders in dispelling myths, translating knowledge of hypertension in pregnancy, and link women to quality care at primary health care facilities ought to be leveraged. Group ANC and women’s groups are potential platforms to improve health literacy, care-seeking attitudes, and use of necessary antenatal and delivery services [21, 22]. Affording access to financial resources that enable women to reach facilities at the community level in Nigeria requires evaluation of the functionality of National Health Insurance Scheme, though could also draw from regional work demonstrating increase in maternal service use among women participating in micro-finance groups [23, 24]. Public policies enhancing primary health care givers’ capacity to manage pre-eclampsia and linking women to a functional referral system that does not rely solely on a family’s financial ability is critical to facilitating care use.

## Conclusion

This study reveals that social-behavioral theory offers useful perspective to understanding care-seeking pathways in maternal health, illuminating the effects of childbearing norms, socio-cultural beliefs and trust in the health system on women’s decisions in northern and southern Nigeria. Where women’s understanding of their susceptibility to and threat of pregnancy complications including pre-eclampsia is limited, the health belief model elucidates opportunities to intervene and improve access to antenatal and emergency obstetric care. Across Nigerian settings, the cues to action identified in our study – as they relate to the benefit of positive health outcomes and greater access to essential emergency services – offer pragmatic implications for investment in (a) programs that enhance context-adapted community awareness-building approaches to promote birth preparedness and social support and (b) development and implementation of national policies emphasizing respectful quality care throughout pregnancy and childbirth.

## Supplementary information

**Additional file 1.** In-depth Interview Guide - woman who experienced pre-eclampsia/eclampsia.

## Data Availability

The dataset analyzed in the current study is not publicly available due to the on-going study terms but will be, upon reasonable request from the corresponding author, after the Ending Eclampsia project is completed.

## Abbreviations

ANC: antenatal care
BP: blood pressure
HBM: Health Belief Model
