# Supplementary material for: Exploring survivor perceptions of pre-eclampsia and eclampsia in Nigeria through the health belief model
Source: BMC Pregnancy Childbirth. 2019 Nov 21;19:431. doi: 10.1186/s12884-019-2582-2 (PMC6873558; doi:10.1186/s12884-019-2582-2)
Supplement: Supplementary file 1 — Additional file 1. In-depth Interview Guide - woman who experienced pre-eclampsia/eclampsia. [file 12884_2019_2582_MOESM1_ESM.docx]

# In-depth Interview Guide - woman who experienced pre-eclampsia/eclampsia

1. How old are you? How old were you when you got married (first time)? How old were you at the time of your first pregnancy?
2. Have you ever attended school/madrasa? What is the highest class you completed?
3. How many living children do you have? What is number/rank of the last child?
4. Are you currently pregnant? If yes, how old is your pregnancy?
5. What is the age of your last child (excluding the index child) When was your last child (child name) born? What was the mode of delivery? Where did you deliver your baby?
6. How far is the nearest government health facility and private hospital or clinic from your home?
7. Did you receive any ANC visit during current/last pregnancy? How many times did you receive ANC checkup in total? At what gestational age did you book this index pregnancy?
8. Where did you go for your first antenatal checkup? Who performed your first antenatal checkup? Did you go to a health facility mainly for antenatal checkup or because you have any other problem(s)? For what problem, if any, did you visit ANC?
9. When you went for ANC please can you tell me what happened during the consultation? *Probe*: did you have your BP measured or urine tested? Did the service providers inform you of the result of any measurement or test? If yes, did the providers explain to you significance of any finding? Can you remember what they told you your BP was?
10. Did the service provider inform you about the possible pregnancy related problems/complications? If yes, please tell me what they said.
11. Did the service provider refer you to another facility for your problem? If yes, for what complications were you referred?
12. Did you receive any treatment for high blood pressure or seizures? If yes, where did you receive the treatment? Did your condition improved/seizure subside after receiving the treatment?
13. Who referred you to this facility? For what problems/complications did you come here? Could you explain what type of problems you were experiencing before coming to this hospital? *Probe:* Did you suffer from severe headache, blurred visions, high blood pressure, swelling feet, or seizures during your pregnancy time?
14. Were you aware that pregnant women may suffer from high blood pressure and or seizures? If yes, tell me what you know about this? Did any service providers tell you about the possibility? *Probe*: what about during ANC?
15. Now, could you briefly describe your experiences at these levels in your effort to seek care for this complication?
16. Family level, including interactions with your spouse and mother in-law?
17. Community members, including religious leaders and traditional service providers?
18. Health care facilities: how were you attended to? Availability of essential drugs, cost of the treatment and outcomes for you and your baby
19. What is your final advice on how you think the problem of high blood pressure and or convulsion in pregnancy can be dealt with at: a) Family level b) Community level c) Health care facility level?
